# Renal Functional Reserve in Acute Kidney Injury Patients Requiring Dialysis

**DOI:** 10.7759/cureus.52901

**Published:** 2024-01-25

**Authors:** Kapil N Sejpal, Priyamvada P S, Madhusudanan Ponnusamy, Naveen K Mattewada, Sreejith Parameswaran, Pranjal Kashiv, Shubham Dubey

**Affiliations:** 1 Nephrology, Jawaharlal Nehru Medical College, Datta Meghe Institute of Higher Education and Research, Wardha, IND; 2 Nephrology, Jawaharlal Institute of Postgraduate Medical Education and Research, Pondicherry, IND; 3 Nuclear Medicine, Jawaharlal Institute of Postgraduate Medical Education and Research, Pondicherry, IND

**Keywords:** rfr, aki, chronic kidney disease (ckd), renal functional reserve, acute kidney injury

## Abstract

The incidence of acute kidney injury (AKI) has increased in the recent past. Patients with AKI have an increased risk of mortality. They are also at increased risk of developing chronic kidney disease (CKD). AKI can lead to irreversible loss of renal function despite complete clinical recovery. Currently, no tools are available to diagnose this subclinical loss of renal function. Renal functional reserve (RFR) can serve as an essential tool for analyzing this subclinical loss of renal function, and patients with loss of RFR post-AKI may be closely followed for the development of CKD. This prospective observational study, conducted at the Department of Nephrology, Jawaharlal Institute of Postgraduate Medical Education & Research (JIPMER), aimed to investigate RFR in 223 patients with AKI requiring dialysis. The study excluded patients with CKD and obstructive uropathy. Methods included RFR assessment three months post-AKI recovery, utilizing technetium-99m (Tc-99m) diethylenetriaminepentaacetic acid (DTPA) plasma clearance during amino acid infusion.

Statistical analyses and logistic regression were applied, receiving ethical approval. Results revealed a high in-hospital mortality rate of 78.02%, associated with elevated Sequential Organ Failure Assessment (SOFA) scores. Among 24 patients with complete AKI recovery, the RFR at three months was 10.06% (interquartile range (IQR) 5.60-20.15), with the measured GFR significantly lower than the estimated glomerular filtration rate (GFR). The study concludes that AKI requiring dialysis is linked to high mortality and emphasizes the predictive value of SOFA scores. Additionally, RFR testing at three months post-recovery provides insights into potential long-term impacts on renal function. This study contributes valuable insights into the prognosis of AKI patients requiring dialysis. It underscores the need for further research on RFR as a diagnostic tool and the lasting consequences of AKI.

## Introduction

There has been an increase in the incidence of acute kidney injury (AKI) globally [1,2]. AKI can have a prolonged detrimental impact on a patient's well-being, which may include heightened occurrences of chronic kidney disease (CKD) and mortality [3,4]. Damage from subclinical or manifest episodes of AKI can result in an irreversible loss of renal mass with a significant impact on renal function [5]. This may be the case despite baseline GFR returning to normal. Renal function reserve (RFR) represents the extent to which kidneys can increase the glomerular filtration rate (GFR) when exposed to various physiological or pathological conditions [6]. GFR may not reduce until half of the nephrons are non-functional or in cases where patients possess a solitary kidney [6,7]. As GFR may remain within the normal range until significant nephron loss has occurred, the RFR test might serve as a sensitive and early method for evaluating functional deterioration in the kidneys and assessing the kidney's capacity for recovery following kidney injury [6,8]. There is a lack of data on RFR after severe dialysis requiring AKI. We hypothesize that patients with clinical recovery from AKI have a reduced RFR.

## Materials and methods

A prospective observational study was conducted at the Department of Nephrology, Jawaharlal Institute of Postgraduate Medical Education and Research (JIPMER), from December 2019 to March 2021. AKI was defined as per the Kidney Disease Improving Global Outcomes (KDIGO) definition for AKI [2,9]. Inclusion criteria included all dialysis-requiring patients with AKI (defined as more than or equal to 1.5 times the increase in baseline creatinine within seven days or more than or equal to 0.3 mg/dl increase from the baseline creatinine within 48 hours or urine output of less than 0.5 ml/kg/hr for six hours). The indication for hemodialysis was decided on clinical grounds. Patients with CKD (eGFR<60 ml/min) and patients with obstructive uropathy were excluded from the study. Patients were followed up three months after the onset of AKI. eGFR was assessed based on the Chronic Kidney Disease Epidemiology Collaboration (CKD-EPI) 2009 equation. RFR was assessed at three months in patients showing complete recovery of AKI (eGFR>60 ml/min and uACR<30). RFR was assessed using diethylenetriaminepentaacetic acid (DTPA) plasma clearance on day zero and DTPA plasma clearance during amino acid infusion on day one.

Technetium-99m (Tc-99m) diethylenetriaminepentaacetic acid (DTPA) was prepared from the DTPA cold kit as per the recommended protocol of the manufacturer (BRIT, Mumbai). The radiochemical purity of the labeled Tc-99m DTPA was measured by paper chromatography and should be more than 95%. Using sterile precautions, 1 mCi of Tc-99m DTPA in approximately 0.5-1 ml was loaded in a syringe. The weight of the loaded radiopharmaceutical was measured using a microbalance (Model: FGB 220, Wensar, Mumbai). A standard syringe containing a similar amount of activity was prepared, weighed, and set aside.

Baseline GFR measurement

On the first day, 1 mCi of Tc-99m DTPA was injected intravenously into the participant. Two venous blood samples of about 3 ml were withdrawn into separate anticoagulated vials at 60 and 180 minutes after injection from a site different from the injection site. The samples were allowed to stand undisturbed for 24 hours so that plasma and cell components could separate.

A standard solution was prepared by dissolving the radiopharmaceutical in a standard syringe in 1000 ml of water 24 hours after injection. Using a micropipette, 1 ml of standard solution and 1 ml of plasma from each of the blood samples were pipetted out. The samples were counted on a gamma counter (Wizard 2, PerkinElmer, USA) for one minute and repeated twice. The GFR was calculated by Russell's two-sample slope intercept method and normalized for body surface area [10].

On the second day, the same procedure was repeated during the infusion of amino acids. The RFR was calculated as a percentage increase in GFR relative to the baseline measurement.

Ethical considerations

The study was approved by the institutional ethics committee of the Jawaharlal Institute of Postgraduate Medical Education and Research, Pondicherry (JIP/IEC/2019/230).

Statistical analysis

The distribution of categorical variables such as gender and body mass index (BMI) was expressed in terms of frequency and percentages. The distribution of continuous variables such as blood pressure, age, and eGFR was expressed in terms of mean with standard deviation or median with interquartile range based on the distribution of the data. The comparison between the eGFR and mGFR was carried out using a paired t-test. The comparison between survivors and non-survivors was done using an independent sample t-test. Binary logistic regression analysis was used to identify independent predictors of mortality associated with AKI.

## Results

A total of 223 patients with AKI who met the inclusion and exclusion criteria were enrolled in the study. Out of which, 174 (78.02%) patients died during the hospital stay. Patients who died during the hospital stay had a higher median Carlson’s co-morbidity index (p = 0.02), Sequential Organ Failure Assessment (SOFA) score (p<0.001), and serum alkaline phosphatase levels (p = 0.002). Hemoglobin levels were significantly lower in patients who died during the hospital stay (p = 0.002). Non-survivors were more likely to have inotropic requirements and mechanical ventilation as compared to the survivors. Males accounted for 71.3% of the study population (n = 159). The mean age of the study population was 47.26 years (SD 15.97). Hypertension was the most common co-morbidity in the study population, present in 56 (25.1%) patients. Median Carlson’s co-morbidity index of the study population was 1 (interquartile range (IQR) 0-3). The most common etiology for AKI was snake envenomation in 44 (19.7%) patients. Of the total study cohort, 170 (76.23%) patients required intensive care unit (ICU) admission at presentation, 119 (53.6%) patients had an inotrope requirement, and 116 (52%) patients were on mechanical ventilation. The most common modality of renal replacement therapy (RRT) received by the patient was sustained low-efficiency dialysis (SLED) in 122 (54.7%) patients. (Table 1).

**Table 1:** Baseline characteristics of the study population CVA: cerebrovascular accident; SLED: sustained low-efficiency dialysis; HD: hemodialysis; PD: peritoneal dialysis; CRRT: continuous renal replacement therapy; SOFA: Sequential Organ Failure Assessment; RRT: renal replacement therapy; SD: standard deviation; IQR: interquartile range

Variable	Value
Age (years)-mean (SD)	47.26 (15.97)
Males-n (%)	159 (71.3%)
Comorbidities-n (%)	
Hypertension	56 (25.1%)
Diabetes mellitus	41 (18.4%)
Cardiovascular disease	18 (8.0%)
Chronic liver disease	17 (7.6%)
Haematological malignancy	13 (5.8%)
Solid organ malignancy	10 (4.5%)
CVA	5 (2.2%)
Carlson’s co-morbidity index-median (IQR)	1 (0-3)
Aetiology-n (%)	
Snake envenomation	44 (19.7%)
Cellulitis/ necrotizing fasciitis	33 (14.8%)
Chronic liver and acute fulminant hepatitis	20 (9%)
Intestinal obstruction /perforation	15 (6.7%)
Acute pancreatitis	15 (6.7%)
Paraquat poisoning	14 (6.2%)
Pneumonia	13 (5.8%)
Cardiorenal syndrome	12 (5.4%)
Poly-trauma and rhabdomyolysis	12 (5.4%)
Multiple myeloma	8 (3.6%)
Liver abscess	7 (3.1%)
Tumour lysis syndrome	7 (3.1%)
Pyelonephritis	7 (3.1%)
Fever with tropical infection	7 (3.1%)
Gastroenteritis	6 (2.7%)
Solid malignancy	2(0.9%)
Urine output at admission(ml)-median (IQR)	700 (300-1100)
Urine output at RRT initiation(ml)-median (IQR)	100 (100-200)
Baseline GFR ml/min-mean (SD)	92.24 (19.87)
ICU admission-n (%)	170 (76.23%)
Inotropic requirement-n (%)	119 (53.4%)
Mechanical ventilation-n (%)	116 (52%)
RRT modality-n (%)	
Intermittent HD	66 (28.3%)
SLED	122 (54.7%)
CRRT	35 (15.7%)
Acute PD	0
No of RRT sessions-median (IQR)	2 (1-3)
SOFA score-median (IQR)	14 (10-15)

Out of the total study population, 24 patients had a complete recovery of AKI, 21 patients had acute kidney disease, and 4 patients developed end-stage renal disease (ESRD). Of these 24 patients, 13 underwent renal reserve study (Table 2). The mean estimated GFR at three months for these patients was 93.33 ml/min (25.38) and the measured GFR was 54.90 ml/min (19.11). The measured GFR was significantly lower than the estimated GFR at 3 months (p<0.001). Median renal reserve at three months was 10.06% (5.60-20.15) (Table 2).

**Table 2:** Renal reserve among survivors of AKI AKI: acute kidney injury; GFR: glomerular filtration rate; IQR: interquartile range; SD: standard deviation

Variable	Value
No of the patients who underwent renal reserve study	13
Estimated GFR at three months ml/min-mean (SD)	93.33 (25.38)
Measured GFR at three months ml/min-mean (SD)	54.90 (19.11)
Renal reserve%-median (IQR)	10.06 (5.60-20.15)
Days from the onset of AKI-median (IQR)	102 (96-148)

## Discussion

AKI is very common in the hospital setting, with incidence ranging from 10-40% [11-13]. AKI is associated with high hospital mortality and worse long-term outcomes, including chronic kidney disease [4,14]. The etiology of AKI varies according to geographical area, with sepsis being the most common cause in developed countries and community-acquired AKI being the most common in developing countries [15]. In our study, the most common reason for AKI was snake envenomation in 44 (19.7%) patients. In our study population, 170 (76.23%) patients required ICU care at enrollment, indicating that most patients were critically ill during registration.

The mortality of the study population was 78.02%, which is higher than the previously described mortality in AKI patients requiring dialysis [16]. Our study had a higher median SOFA score and more patients requiring inotropes and mechanical ventilation, which might have accounted for the increased mortality observed in this population.

The limitations of the study were reduced follow-up due to the COVID-19 pandemic and a limited number of patients due to high mortality among the study participants. Twenty-four patients had complete recovery from AKI, out of which 13 underwent renal functional reserve study. The renal reserve represents the capacity of the kidneys to augment their basal glomerular filtration rate (GFR) after a protein overload or amino acid infusion. The normal renal reserve of the general population is 20-25%. In conditions of stress like pregnancy, diabetic nephropathy, hypertension, kidney donors, or solitary kidneys, the kidneys utilize this renal reserve to maintain the normal GFR. In patients who have recovered from AKI, the eGFR may appear to be expected, but this may be at the cost of loss of renal reserve. The renal reserve test can be utilized as a sensitive and early method to evaluate the decline in kidney function and assess the kidney's capacity for recovery after a kidney injury [6]. Data on renal reserve following acute kidney injury has been sparse. In a pediatric study of patients with post-streptococcal glomerulonephritis (PSGN), renal reserve was significantly lower after recovery than the controls [17]. In another clinical study of children recovered from hemolytic uremic syndrome, it was found that the renal reserve of the children who recovered was significantly lower than the controls. At a follow-up of eight years, only the patients with reduced renal reserve developed proteinuria [18].

In a study done on patients who developed AKI post-cardiac surgery, renal reserve was measured pre-surgery and three months post-surgery. This study found that renal reserve significantly decreased three months post-cardiac surgery in patients who developed AKI, even though the eGFR was normal [19]. The median renal reserve in our study population was 10.06% (5.60-20.15).

## Conclusions

AKI requiring dialysis was associated with a high rate of hospital mortality. A high SOFA score on admission was independently associated with higher mortality. The median RFR at three months was 10.06% (IQR 5.60-20.15) in patients who showed complete recovery of AKI. The measured GFR was significantly lower at three months of follow-up as compared to the estimated GFR in patients who showed complete recovery of AKI.

## References

[1] Kam Tao Li P, Burdmann EA, Mehta RL (2013). Acute kidney injury: global health alert. J Nephropathol.

[2] Makris K, Spanou L (2016). Acute kidney injury: definition, pathophysiology and clinical phenotypes. Clin Biochem Rev.

[3] Soto K, Campos P, Pinto I, Rodrigues B, Frade F, Papoila AL, Devarajan P (2016). The risk of chronic kidney disease and mortality are increased after community-acquired acute kidney injury. Kidney Int.

[4] Lafrance JP, Miller DR (2010). Acute kidney injury associates with increased long-term mortality. J Am Soc Nephrol.

[5] Ronco C, Rosner MH (2012). Acute kidney injury and residual renal function. Crit Care.

[6] Sharma A, Mucino MJ, Ronco C (2014). Renal functional reserve and renal recovery after acute kidney injury. Nephron Clin Pract.

[7] Gounden V, Bhatt H, Jialal I (2023). Renal Function Tests. StatPearls [Internet].

[8] Fuhrman DY (2021). The role of renal functional reserve in predicting acute kidney injury. Crit Care Clin.

[9] Goyal A, Daneshpajouhnejad P, Hashmi MF, Bashir K (2023). Acute Kidney Injury. StatPearls [Internet].

[10] Russell CD, Bischoff PG, Kontzen FN (1985). Measurement of glomerular filtration rate: single injection plasma clearance method without urine collection. J Nucl Med.

[11] Kellum JA, Lameire N (2013). Diagnosis, evaluation, and management of acute kidney injury: a KDIGO summary (Part 1). Crit Care.

[12] Susantitaphong P, Cruz DN, Cerda J, Abulfaraj M, Alqahtani F, Koulouridis I, Jaber BL (2013). World incidence of AKI: a meta-analysis. Clin J Am Soc Nephrol.

[13] Wang HE, Muntner P, Chertow GM, Warnock DG (2012). Acute kidney injury and mortality in hospitalized patients. Am J Nephrol.

[14] Doyle JF, Forni LG (2016). Acute kidney injury: short-term and long-term effects. Crit Care.

[15] Rewa O, Bagshaw SM (2014). Acute kidney injury-epidemiology, outcomes and economics. Nat Rev Nephrol.

[16] Vikrant S, Gupta D, Singh M (2018). Epidemiology and outcome of acute kidney injury from a tertiary care hospital in India. Saudi J Kidney Dis Transpl.

[17] Cleper R, Davidovitz M, Halevi R, Eisenstein B (1997). Renal functional reserve after acute poststreptococcal glomerulonephritis. Pediatr Nephrol.

[18] Dieguez S, Ayuso S, Brindo M, Osinde E, Cánepa C (2004). Renal functional reserve evolution in children with a previous episode of hemolytic uremic syndrome. Nephron Clin Pract.

[19] Husain-Syed F, Ferrari F, Sharma A (2019). Persistent decrease of renal functional reserve in patients after cardiac surgery-associated acute kidney injury despite clinical recovery. Nephrol Dial Transplant.

